# Persistent gaps in skin cancer prevention

**DOI:** 10.1016/j.eclinm.2026.104006

**Published:** 2026-05-27

**Authors:** eClinicalMedicine

In May, Skin Cancer Awareness Month offers a welcome reminder about the risk and burden of skin cancer, the importance of sun protection, and the need for early detection to save lives. Despite the increasing recognition and knowledge of the disease, incidence persists and continues to rise in many settings alongside inequities in diagnostics, exposure, and protection. Skin cancers are the most common group of cancers diagnosed worldwide, with more than 1.5 million new cases estimated globally in 2022 according to most-recent estimates. Newly diagnosed invasive melanoma cases have shown a 46.6% increase in annual incidence over the past decade in the USA and, looking ahead, modelling has estimated cases to increase globally by more than 50% by 2040 to 510,000 per year, with deaths from melanoma estimated to increase by approximately 68%, from 57,000 in 2020 to 96,000 in 2040. The continued rise in skin cancer suggests that awareness has not yet translated into sustained prevention at the population level and, although there is recognisable effort, crucial gaps persist resulting in the amounting burden.

Skin cancer is among the most preventable malignancies, so this raises the question as to why the prevalence is so high. The main risk factor, ultraviolet (UV) radiation exposure, is well known and there are effective protective measures such as sunscreen. However, various factors contribute to the burden of skin cancers, including, for example, aging populations, in turn leading to greater cumulative UV radiation exposure for individuals; unequal access to protective measures; behavioural patterns and societal norms around tanning; and sunbed use, with a so-called long tail effect from the cumulative, delayed, and often permanent damage which may not lead to cancer until many years after the exposure occurred. Climate change also plays a role, with rising temperatures, depletion of the ozone layer, and increased sun exposure resulting in extreme heatwaves which have been shown to be a major risk factor for skin cancer. Regions with historically low UV exposure might now need to implement measures to combat the increased risk. Despite awareness of the risk factors, challenges arise from inconsistent sun protection, late presentation with symptoms, inequalities in access to diagnosis across different settings, and low perceived risk in specific populations, such as young people or those with darker skin tones.

To further close the gaps in prevention, structural change is required. Population-level interventions have been shown to work, for example in Australia, where sustained, multicomponent campaigns combining education and school-based policies, and the total legislative ban on commercial indoor tanning beds in 2016, have coincided with stabilisation or decline in melanoma incidence, particularly among younger cohorts. In other countries, progress is not so clear. Regulatory tools—such as taxes, restrictions, or outright bans on indoor tanning; standards for UV-protective clothing; and reduced taxes on sunscreen—remain underused in many countries. If formally implemented, these regulations could reduce exposure at scale. Schools and workplaces (especially for those with outdoor occupations) represent a crucial opportunity to reduce exposure and adequate sun protection must be provided in these contexts. Initiatives to curb climate change and depletion of the ozone layer are also vital to reduce the risk of skin cancers. Awareness campaigns frequently rely on individual responsibility, but this approach is limited in its effectiveness. Public health messaging that relies solely on warnings is not enough; for real impact, shifts in policy are required and governments must take more responsibility to tackle the rise in cases.

Early detection of skin cancer lesions is crucial to reduce mortality, but patients frequently present at advanced stages. Opportunistic detection is most common and organised diagnostic approaches are rare, with evidence for asymptomatic screening showing no clear benefit. So, what more can be done? Risk stratification, identifying individuals with family history, genetic susceptibility, or high cumulative UV radiation exposure could lead to greater yield. Early evidence has also shown potential of using a neural network-based explainable artificial intelligence (AI) model with 2D facial image inputs as an alternative to risk factors alone for identifying individuals at high risk of skin cancer. Implementation of an AI-based mobile health application for skin cancer detection to be used by patients themselves or as a diagnostic tool for general practitioners has also shown feasibility. These tools might have potential to lead to earlier detection while reducing pressures on health-care providers but to ensure accuracy and reliability, further evaluation, more research, and additional tools are still needed. At the same time, referral pathways require investment for greater accessibility and reduced waiting times to ensure that suspected cancers are assessed promptly.

Inequalities are another area that require attention. Skin cancer in patients with darker skin tones is often diagnosed in later stages, when it is more difficult to treat, and these patients are less likely to survive melanoma than those with lighter skin tones. More needs to be done to close this gap and ensure identification is not delayed in specific patient groups. Socioeconomic factors also shape exposure and protection, for example limited availability and high cost of sunscreen might mean those from more deprived socioeconomic backgrounds are less protected and at a greater risk of developing skin cancer. Furthermore, access to dermatology services varies geographically and does not always reach those at highest risk. While AI might be able to assist in early detection and triage, this carries concerns about validation across diverse skin types and risks of widening disparities if deployed without attention to access.

Although there have been advances in therapies, prevention remains preferable. Awareness must be matched by policy and practice for greater impact. Primary prevention of skin cancer must be strengthened through regulation and equitable access to protective measures, integrating early detection into routine care, ensuring measures serve all skin types and communities, and implementation research for further preventive measures. Most skin cancers are preventable, and there must be commitment to policy, clinical, and societal changes to reduce cases in reality.

